# Executive self-compassion, strategic inertia, and corporate performance

**DOI:** 10.3389/fpsyg.2026.1775552

**Published:** 2026-04-10

**Authors:** Ting Yu, Ning Liu, Yixin Wang

**Affiliations:** 1 School of Business and Law, Sanjiang University, Nanjing, China; 2 School of Management, Nanjing University of Posts and Telecommunications, Nanjing, China; 3 School of Business, Nanjing University, Nanjing, China

**Keywords:** common humanity, executive, firm performance, mindfulness, self-kindliness, strategic inertia

## Abstract

Based on upper echelons theory, this paper analyzes the influencing mechanism between executives’ self-compassion attributes (common humanity, self-kindliness, mindfulness,) and firm performance, and the mediator effect of firm strategic inertia. The empirical analysis of the sample data of 332 valid questionnaires from the architectural design industry in China shows that: (1) the common humanity and mindfulness of executives positively promote firm strategic inertia; (2) the self-kindliness dimension of executives inhibits firm strategy inertia; (3) strategic inertia hinders the development of firm performance in highly variable industries; (4) strategic inertia plays a partial mediating role between executive self-compassion attributes and firm performance.

## 1 Introduction

In dynamic environments, a firm’s ability to overcome strategic inertia—a tendency to persist with established strategic patterns—is critical in sustaining competitive advantage. In the field of strategic management, elucidating the formation mechanisms of strategic inertia has garnered growing scholarly attention owing to its pivotal role in shaping organizational longevity. While existing studies have predominantly focused on executives’ demographic characteristics (e.g., gender, tenure, and educational background) or explicit governance factors (e.g., compensation and succession) (Carpenter, 2000; Finkelstein and Hambrick, 1990; Nwafor et al., 2025; Wiersema and Bantel, 1992; Xue et al., 2022; Zhang and Rajagopalan, 2010), little scholarly attention has been paid to how executives’ psychological characteristics shape strategic inertia. The upper echelons theory posits that executives’ characteristics influence organizational outcomes by shaping their attention, interpretation, and sensemaking of strategic information (Hambrick, 2007), thereby providing a direct analytical framework for unraveling how psychological traits affect strategic choices. According to the upper echelons theory, observable proxies such as demographic attributes are merely surface-level indicators, which fall short of illuminating the core cognitive and affective drivers within the “black box” of strategic decision-making. The intrinsic, stable psychological disposition of top managers fundamentally underpins their cognitive frameworks and subsequent strategic choices (Hambrick, 2007). Recent studies continue to affirm and extend this core premise, with a particular focus on the strategic role of executives’ psychological and behavioral characteristics in complex environments. (e.g., Guo et al., 2025; Tian et al., 2025; Tuggle et al., 2024; Yu et al., 2025).

Among various psychological traits, self-compassion offers a particularly fitting conceptual lens for elucidating strategic inertia because of its multidimensional structure that integrates cognitive and emotional regulation. Unlike other personality traits (e.g., the Big Five) or psychological states (e.g., psychological capital), self-compassion encompasses three dimensions—common humanity, self-kindness, and mindfulness—that can systematically influence the three key cognitive stages in strategic decision-making information processing: selective attention (what they pay attention to), information interpretation (how they interpret incoming information), and sensemaking (how they generally make sense of the organization’s surroundings) (Hambrick, 2007). While each dimension holistically affects the cognitive process, its influence follows a dominant pathway: common humanity primarily directs attention toward relationship-maintenance information; self-kindness enhances openness to challenging information by bolstering psychological safety; and mindfulness, characterized by non-judgmental present-moment awareness, shapes how executives integrate information and construct environmental meanings. Collectively, these dimensions form a dynamic cognitive filtering system that determines a firm’s reliance on established strategies. Therefore, focusing on the construct of self-compassion helps to penetrate surface-level variables and reveal the psychological formation mechanism of strategic inertia through an integrated cognitive–affective pathway, thereby addressing the current gap in micro-level psychological drivers.

Building on this rationale, this study investigates whether and the manner in which executives’ self-compassion influences corporate strategic inertia, and ultimately, firm performance by shaping their cognitive filtering mechanisms. We postulate that the three dimensions of self-compassion (common humanity, self-kindness, and mindfulness) systematically enhance or inhibit strategic inertia by influencing executives’ selective attention, information interpretation, and sensemaking during strategic decision-making, thereby having a domino effect on organizational performance.

To test this theoretical framework empirically, this study focuses on the architectural design industry in China. This context was chosen because it represents a setting in which the underlying mechanisms are likely to be salient. First, as a highly knowledge-intensive service industry, its competitive advantage hinges critically on the strategic foresight and innovative decision-making of executives, making the impact of their psychological traits more observable. Second, the industry operates under a persistently high environmental dynamic characterized by frequent policy shifts, rapid technological iterations, and volatile market demand. Under such conditions, the performance consequences of strategic inertia are amplified, offering an ideal testing ground for examining the hypotheses of this study.

## 2 Theoretical foundation and research hypotheses

Strategic inertia refers to the persistence of a firm’s strategic direction and patterns, reflecting its resistance to change when confronted with external shifts (Lian and He, 2015). Existing research has identified its antecedents such as structural constraints, resource stocks, organizational routines, and deeply entrenched managerial beliefs (Bingham and Davis, 2012; Matthew and Edward, 2001; Stryker and Burke, 2000). Nevertheless, research on the underlying psychological characteristics of executives—the core agents of strategic decision-making—remains scarce. To address this gap and unpack how executives’ inner traits translate into strategic persistence, this study is grounded in the upper echelons theory. Grounded in the upper echelons theory, executives are not perfectly rational actors, and their strategic choices are constrained by bounded rationality. Particularly, complex internal and external strategic information is not objectively “knowable” but must be interpreted (Hambrick, 2007). Executives’ psychological traits shape their selective attention, interpretation, and sensemaking of information—a cognitive filtering mechanism that ultimately influences strategic outcomes at the organizational level (Hambrick and Mason, 1984). Consequently, unpacking the “black box” of how executive traits affect strategic decisions necessitates a deeper evaluation of their underlying psychological and cognitive processes.

Conventional research has often employed broad traits such as the Big Five personality factors, core self-evaluations, or narcissism to predict executive behavior. While these constructs offer wide coverage, their generalized structures often fall short of revealing the specific, organically linked cognitive–affective processes underlying strategic decision-making. For instance, the Big Five dimension of conscientiousness may manifest either as adherence to established plans or as engagement in new exploratory initiatives (Judge et al., 2002), creating theoretical ambiguity in its relationship to strategic outcomes. Similarly, narcissism, characterized by inflated self-views and dominance (Rosenthal and Pittinsky, 2006), may partially elucidate strategic risk-taking driven by overconfidence, but fails to systematically account for the inner psychological regulatory mechanisms that sustain or adjust strategic direction in the face of setbacks, ambiguity, and social pressures. In contrast, self-compassion—as an integrative psychological construct encompassing self-kindness, common humanity, and mindfulness—directly maps onto the cognitive-affective regulation processes crucial for strategic adaptation. Its relevance in organizational settings, particularly for leadership and adaptation, is gaining empirical support (Ashford et al., 2022; Dodson and Heng, 2022). Its three dimensions can be theoretically linked to the key cognitive filters (attention, interpretation, sensemaking) outlined by upper echelons theory, offering a more precise mechanism to explain strategic persistence or change. Thus, self-compassion demonstrates greater theoretical focus and explanatory precision in the context of strategic decision-making than classic leadership traits.

### 2.1 Executive common humanity and strategic inertia

Common humanity refers to the cognitive tendency to perceive one’s experiences as part of the universal human condition, with its core in transcending egocentrism and establishing psychological connectedness with others (Neff, 2003). In organizational contexts, this trait is often reflected in the altruistic orientation, empathetic capacity, and motivation of executives to maintain relationships (Ashton and Lee, 2021). While some studies suggest that common humanity helps build a psychologically safe climate of trust and collaboration, thus promoting routine innovation and voice behavior within teams (Giancola et al., 2021), high levels of common humanity may also reinforce an organization’s reliance on existing strategies through a relationship–preservation–oriented cognitive filtering bias. This mechanism can be explained by integrating the upper echelons theory with social information processing theory.

First, according to the upper echelons theory, executive characteristics shape the priority order of attention allocation (Hambrick, 2007). The strong empathy and altruistic motivation inherent in high common humanity may lead executives in complex and volatile strategic decision-making situations to unconsciously prioritize social information related to “maintaining team harmony,” “avoiding employee resistance,” and “preserving relational stability.” For instance, LePine and Van Dyne (2001) established that individuals high in agreeableness—a dimension that closely overlaps with common humanity—tend to avoid challenging information or actions that could provoke interpersonal conflict or disrupt harmony. This aligns with the concept of “commitment to the status quo” (CSQ), where a desire to preserve social harmony and stability can become a cognitive barrier to recognizing the need for change (Chiu et al., 2020).

Conversely, critical economic and competitive signals that may trigger internal conflicts, such as industry-disruptive trends or structural adjustment pressures, are prone to neglect or downplay. This relationship–maintenance-oriented attention configuration constitutes an input bias at the forefront of strategic decision-making.

Second, drawing on social information processing theory, which serves as a complementary lens to the upper echelons theory by detailing how individuals’ attitudes and behaviors are shaped by their social information environment (Salancik and Pfeffer, 1978), we can further specify the mechanism at the interpretation stage. This theory elucidates that executives with high common humanity not only focus on relational information but are also inclined to seek and adopt information from specific social contexts that align with their relational goals. Their cognitive frameworks are more inclined to interpret the meaning of information from a socioemotional perspective. During decision-making, they may more actively seek and adopt opinions from internal networks that favor maintaining the status quo, while subconsciously subjecting external signals advocating radical change to a “harmonizing” reinterpretation, aligning with their intrinsic motivation to preserve organizational community stability. This process essentially reinforces the organizational narrative favoring the status quo through selective social information processing and sensemaking, thereby collectively cementing reliance on existing strategies.

In summary, during the attention and interpretation stages of strategic decision-making, increased common humanity systematically weakens executives’ objective scrutiny of the urgency and necessity of change by directing attention toward internal social costs and shaping an information environment biased toward relational stability. Even when the external environment sends strong signals for change, the organization may respond slowly because of the cognitive prioritization of internal relationship preservation, thereby exhibiting strategic inertia. Thus, we propose the following:

Hypothesis 1: Executive common humanity is positively associated with strategic inertia.

### 2.2 Executive self-kindness and strategic inertia

According to the upper echelons theory, characteristics of executives influence strategic choices by influencing the interpretation of information, a pivotal cognitive stage in the strategic decision-making process (Hambrick, 2007). Self-kindness refers to the tendency to be understanding and supportive, rather than self-critical, when confronted with personal shortcomings or difficult situations. Its inhibitory effect on strategic inertia is achieved by refining the cognitive and affective processes underlying executives’ interpretation of information.

Particularly, self-kindness helps buffer against negative emotions associated with stressful or frustrating events (Ferradás Canedo et al., 2022). When encountering ambiguous, contradictory, or cognition-challenging strategic information, such as signals of performance decline or sudden shifts in the competitive landscape, executives who exhibit high self-kindness, owing to their self-acceptance and tolerance, can effectively mitigate defensive psychological reactions triggered by potential threats of failure. This low-defensiveness state enables them to reduce interpretive biases driven by the need to maintain “self-correctness” when processing such information; for example, they are less likely to attribute negative information to external uncontrollable factors or to rationalize failures of existing strategies. Recent research on leader self-compassion supports this, showing that it reduces defensiveness and fosters a more open, learning-oriented approach to challenges (Ashford et al., 2022). Self-kindness positively enhances an individual’s sense of self-efficacy (Liao et al., 2021). Executives with higher self-efficacy possess greater confidence in their ability to handle challenges and address novel problems; consequently, when interpreting complex or threatening information, they tend to adopt an open, exploratory cognitive frame, viewing it as a “challenge” to be managed and learned rather than a “threat” to be avoided. This interpretive inclination directly weakens the cognitive motivation to distort the meaning of information to preserve the status quo.

Thus, viewed through the cognitive pathway of the upper echelons theory, high self-kindness reduces defensive biases and increases openness in the information-interpretation stage, allowing executives to interpret signals for change more objectively and flexibly, thereby diminishing the strategic path dependence arising from cognitive rigidity. This constitutes the core cognitive mechanism by which self-kindness inhibits strategic inertia. Therefore, we proposed the following:

Hypothesis 2: Executive self-kindness is negatively associated with strategic inertia.

### 2.3 Executive mindfulness and strategic inertia

Mindfulness is defined as “the conscious state of maintaining purposeful, non-judgmental attention to present-moment experiences” (Kwee, 1995). According to the upper echelons theory, executives rely on sensemaking to interpret the organizational environment and form strategic judgments (Hambrick, 2007). The “non-judgmental awareness of present-moment experience” cultivated by mindfulness may exert a dual effect on sensemaking at the strategic level: while it enhances the detection of immediate, concrete information, it can also systematically shape how executives define and interpret environmental signals.

Information is often ambiguous, contradictory, or overloaded in dynamic and complex strategic contexts. First, from an attention-based perspective, executive attention is scarce (Ocasio, 1997). The sustained focus on “present-moment” tasks and performance feedback reinforced by mindfulness may lead to an over-allocation of cognitive resources toward processing immediate, verifiable, and structured information, thereby relatively neglecting those ambiguous, weak, yet potentially transformative long-term or unstructured environmental signals. This convergence of attention toward operational immediacy can reduce sensitivity to long-term trends such as shifts in industry structure or technological paradigm transitions, resulting in strategic far-sightedness. Second, by integrating the need for cognitive closure theory, under the pressure of strategic decision-making characterized by high uncertainty and information overload, the tendency toward “non-judgmental acceptance” inherent in mindfulness may convince executives to accept and adhere to interpretations within existing cognitive frameworks rather than proactively subject them to fundamental questioning. To reduce cognitive load and decision anxiety, they may increase reliance on familiar causal schemas validated by past successes to interpret new information, thereby falling into a “competency trap” (Levinthal and March, 1993). This is since such behavior generates strong selective perception biases by causing strategic decision-makers to ignore novel and divergent environmental information (Hortovanyi et al., 2021). This uncritical maintenance of existing sensemaking frameworks inhibits the in-depth exploration of disruptive information and the reconstruction of environmental meaning, thereby reinforcing dependence on established strategic paths.

In summary, executives’ mindfulness may collectively lead to a more inward-looking and rigid understanding of environmental changes by directing attention to immediate feedback and reinforcing the preservation of existing cognitive frameworks. This makes it difficult for the organization to promptly identify strategic inflection points or reconfigure competitive logic, often delaying strategic adjustment until influenced by compelling feedback, such as a significant performance decline. Accordingly, we proposed the following:

Hypothesis 3: Executive mindfulness will be positively associated with strategic inertia.

### 2.4 Strategic inertia and firm performance

In today’s dynamic business environment characterized by rapidly shifting competitive and technological contexts, strategic inertia can adversely affect firm performance through several mechanisms. First, it can lock firms into outdated processes and resource configurations, thereby negatively impacting their performance (Back et al., 2020). Second, organizations with strong inertia typically exhibit sluggish responses to external signals, structural rigidity, and insufficient learning capacity, making it difficult for them to promptly adjust their strategic positioning in a rapidly changing environment. Hitt et al. (1998) emphasize that in the context of hypercompetition and frequent technological disruptions, a firm’s survival and growth depend directly on its ability to respond and learn quickly in the face of discontinuous change, whereas inertia fundamentally erodes this adaptive foundation. Finally, the maintenance of inertia often relies on established organizational routines, power structures, and interest patterns, which can reinforce departmental barriers, impede information flow, and prolong decision-making processes, thereby significantly increasing internal coordination and agency costs (Williamson, 1985). In the long run, this internal friction reduces the overall organizational effectiveness and inhibits the efficiency with which market opportunities can be captured. Accordingly, we proposed the following:

Hypothesis 4: Strategic inertia negatively correlates with firm performance.

### 2.5 Mediating role of strategic inertia

According to the upper echelons theory, executives operate under bounded rationality and selective perception. Their interpretations of strategic information and subsequent strategic choices are shaped by their cognitive frameworks and values. Building on this logic, we argue that executives’ self-compassion influences how they acquire, filter, and interpret information, which, in turn, affects the degree of strategic inertia. As inertia has performance implications, it is likely to serve as a mediating mechanism between executive self-compassion and firm outcomes (Hambrick and Mason, 1984).

First, executive self-compassion influences strategic inertia through a cognitive filtering mechanism. The three dimensions of self-compassion—common humanity, self-kindness, and mindfulness—systematically shape how executives process strategic information. Common humanity directs the focus of selective attention toward relationship-maintaining information; self-kindness enhances openness in information interpretation, reducing defensive distortion of challenging information; and mindfulness moderates the breadth and depth of sensemaking, influencing how environmental signals are integrated. The cognitive outputs filtered through these traits directly manifest in whether executives tend to preserve existing strategic paths or promote strategic change—that is, the intensity of strategic inertia at the organizational level. Therefore, self-compassion influences strategic inertia through cognitive processes.

Second, as an organizational behavioral pattern, strategic inertia directly affects a firm’s adaptability and resource allocation efficiency. Strategic inertia reflects an organization’s tendency to maintain its existing strategic direction and resource configuration when facing internal and external changes. High inertia inhibits the speed of organizational response to external opportunities and threats; locks resources into outdated technologies, products, or processes; and hinders innovation and adjustment (Tripsas and Gavetti, 2000). In a dynamic competitive environment, this rigidity can cause firms to miss market opportunities, increase internal coordination costs, and reduce operational flexibility, thereby directly and negatively affecting performance.

Finally, strategic inertia serves as a conduit to transmit the influence of executive traits on performance, thereby constituting a complete mediation pathway. Viewed through the integrated chain of “trait → cognition → behavior → outcome,” executives’ self-compassion first shapes whether an organization favors strategic persistence or strategic change—that is, the level of strategic inertia—by influencing their cognitive filtering mechanisms. Strategic inertia, as a stable organizational behavioral pattern, determines a firm’s adaptive capacity and the efficiency of its resource allocation in dynamic environments, ultimately manifesting as differences in performance levels. Strategic inertia plays the pivotal mediating role of a key bridge: it translates psychological traits at the individual executive level into observable organizational behavior and ultimately transmits its effects to organizational performance. Therefore, we proposed the following:

Hypothesis 5: Strategic inertia mediates the relationship between executive self-compassion and firm performance.

**Figure 1:**
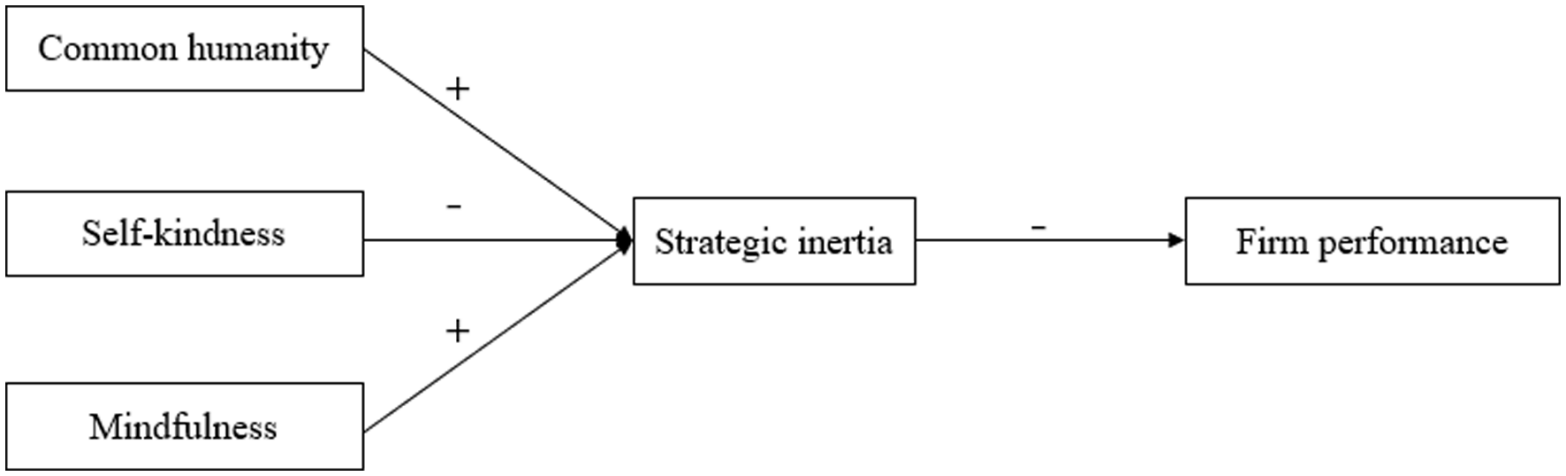
Theoretical model.

The model of this study is shown in Figure 1.

## 3 Research design

### 3.1 Sample and data collection

This study focuses on individual senior executives in China’s architectural design industry. Given the high access barriers and contact constraints associated with the executive population, random sampling was not feasible in practice. Therefore, with the assistance of industry associations, a snowball sampling method was employed for data collection. The specific survey procedure was as follows: First, eligible executives were contacted through industry associations and partner firms and invited to complete the questionnaire. Subsequently, a brief note was attached at the end of the questionnaire inviting respondents to recommend other qualified senior executives to participate in the survey. Data were collected through a combination of online platforms (e.g., Tencent Questionnaire and Wenjuanxing APP) and limited on-site distribution. To mitigate common method bias, a two-wave survey design with a time lag of over 1 month was adopted: the first wave measured executives’ self-compassion, firm characteristics, strategic inertia, and other variables, while the second wave collected data on firm performance and related information. All returned questionnaires were matched and screened using information such as company name and respondent position to ensure data validity.

A total of 448 questionnaires were distributed; after excluding invalid responses (e.g., incomplete or low-quality entries), 332 valid questionnaires were retained, yielding a valid response rate of 74.1%. Sample characteristics are summarized in Table 1.

**Table 1:** Distribution characteristics.

Variable	Category	Count (Persons)	Percentage
Firm size	1–20	38	11.4%
	21–40	21	6.3%
	41–60	16	4.8%
	61–80	14	4.2%
	81–100	35	10.5%
	>100	208	62.7%
Firm age	1–6 years	56	16.9%
	6–12 years	48	14.5%
	>12 years	228	68.7%

### 3.2 Measures

All constructs, except for control variables, were measured using a 5-point Likert scale. All English measurement instruments were translated into Chinese following the back-translation procedure (Brislin, 1970).

#### 3.2.1 Executive self-compassion

We measured executive self-compassion with reference to Neff (2003), using a scale comprising the three dimensions of common humanity, self-kindness, and mindfulness. The common humanity dimension included 3 items (e.g., “When things are going badly for me, I see the difficulties as part of life that everyone goes through”), with a reliability of 0.868. The mindfulness dimension included 3 items (e.g., “When I fail at something important to me, I try to keep things in perspective”), with a reliability of 0.903. The self-kindness dimension included 4 items (e.g., “I’m kind to myself when I’m experiencing suffering”), with a reliability of 0.904. We further conducted confirmatory factor analysis (CFA) on the three dimensions of the independent variable, comparing the hypothesized three-factor model with several alternative models with merged factors. Specifically, we compared the three-factor model (with self-kindness, common humanity, and mindfulness as three distinct latent variables), a two-factor model (merging self-kindness and common humanity into one factor), and a single-factor model (loading all items onto one latent variable). The three-factor model demonstrated a good fit (χ^2^ = 134.506, df = 24, TLI = 0.920, CFI = 0.957, SRMR = 0.037). In contrast, both the two-factor model (χ^2^ = 294.200, df = 26, TLI = 0.821, CFI = 0.896, SRMR = 0.113) and the single-factor model (χ^2^ = 439.767, df = 27, TLI = 0.734, CFI = 0.841, SRMR = 0.109) showed significantly poorer fit. Chi-square difference tests confirmed that the three-factor model fit significantly better than both the two-factor model (Δχ^2^ = 159.694, *p* < 0.001) and the single-factor model (Δχ^2^ = 305.261, p < 0.001). These results provide strong empirical support for the discriminant validity of the three dimensions within the executive decision-making context examined in this study.

#### 3.2.2 Firm performance

Prior research notes that organizational performance is a multidimensional and context-dependent construct that is difficult to comprehensively capture with a single indicator (Richard et al., 2009). Firms operating under different market conditions, with varying resources and strategic goals, often have significantly different performance standards. Therefore, in the absence of perfectly comparable objective data, perceptual performance evaluations by managers are widely used in organizational and strategic management research to measure a firm’s relative performance within its competitive environment, thereby enhancing the comparability of performance assessments across firms of different sizes (Leicht-Deobald et al., 2021). Drawing on the work of Auh et al. (2019), this study adopts a perceptual performance measurement approach to assess firm performance. Specifically, responding managers were asked to evaluate their firm’s performance in the following three areas: firm profitability, sales growth capability, and customer retention capability. These three indicators reflect key performance dimensions pertaining to financial results, market expansion, and customer relationship maintenance, respectively. The scale demonstrated a reliability of 0.902.

#### 3.2.3 Strategic inertia

Strategic inertia was measured with reference to Lian and He (2015), using 6 items (e.g., “Compared with the previous year, the revenue proportion of each business segment to total revenue has not changed”). The scale’s reliability was 0.855.

#### 3.2.4 Control variables

Following prior research, we controlled for executive age, firm size, and firm age.

## 4 Data analysis and hypothesis testing

### 4.1 Preliminary analysis

As shown in Table 2, all constructs in this study met the criteria of CR > 0.70 and AVE > 0.50, indicating satisfactory internal consistency and convergent validity. Table 3 demonstrates that the square roots of the AVEs for all constructs were greater than the correlations between that construct and any other construct, supporting the discriminant validity of the measures. Additionally, Harman’s single-factor test was used to assess common method bias (CMB). The results extracted five factors, with the first factor accounting for 29.747% of the variance, which is below the 50% threshold, suggesting that common method bias is not a serious concern in this study (Bunjak et al., 2022).

**Table 2:** Reliability and validity statistics.

Variables	Cronbach’s alpha	AVE	CR
Common humanity	0.868	0.529	0.771
Self-kindness	0.904	0.701	0.903
Mindfulness	0.903	0.709	0.878
Firm Performance	0.902	0.766	0.907
Strategic Inertia	0.855	0.557	0.877

**Table 3:** Pearson correlation coefficients between variables.

Variables	Mean	SD	1	2	3	4	5	6	7	8
1 Executive Age	2.262	0.782								
2 Firm Size	4.840	1.802	−0.114*							
3 Firm Age	2.518	0.767	0.121*	0.377***						
4 Self-kindness	3.705	0.707	−0.138*	0.094	0.244***	(0.837)				
5 Common Humanity	3.852	0.714	0.248***	−0.014	0.412***	0.413***	(0.727)			
6 Mindfulness	3.906	0.809	0.057	0.037	0.283***	0.496***	0.553***	(0.842)		
7 Strategic Inertia	3.245	0.776	0.130*	0.118*	0.081	−0.201***	0.189**	0.196***	(0.746)	
8 Firm Performance	3.780	0.802	−0.065	−0.153**	0.025	0.257***	0.247***	0.051	−0.264***	(0.875)

^*^p < 0.05, ^**^p < 0.01, ^***^p < 0.001. The values in parentheses along the diagonal are the square roots of the AVE for each construct.

### 4.2 Correlation coefficients analysis

Pearson correlation analysis was conducted using the mean scores of the latent variables. The results, presented in Table 3, provide preliminary evidence. A significant negative correlation was found between strategic inertia and firm performance (*r* = −0.264, *p* < 0.001), indicating that stronger strategic inertia is associated with poorer firm performance, which is consistent with Hypothesis 4. Regarding the dimensions of executive self-compassion, both common humanity (*r* = 0.189, *p* < 0.01) and mindfulness (*r* = 0.196, *p* < 0.001) showed significant positive correlations with strategic inertia, supporting Hypotheses 1 and 3, respectively. In contrast, the self-kindness dimension (*r* = −0.201, *p* < 0.001) was significantly negatively correlated with strategic inertia, consistent with Hypothesis 2.

### 4.3 Hypothesis testing

We tested the research hypotheses using linear regression analysis. The results are presented in Table 4. Model 4 shows that common humanity has a significant positive effect on strategic inertia (*β* = 0.247, SE = 0.073, *p* < 0.01), supporting Hypothesis H1. Self-kindness has a significant negative effect on strategic inertia (*β* = −0.485, SE = 0.066, *p* < 0.001), supporting Hypothesis H2. Mindfulness has a significant positive effect on strategic inertia (*β* = 0.286, SE = 0.061, *p* < 0.001), supporting Hypothesis H3. Model 1 indicates that strategic inertia has a significant negative effect on firm performance (*β* = −0.252, SE = 0.055, *p* < 0.001), supporting Hypothesis H4.

**Table 4:** Regression analysis results.

Variables	Dependent variable: firm performance									Dependent Vvariable: strategic inertia		
	Model 1			Model 2			Model 3			Model 4		
	*β*	*SE*	VIF	*β*	*SE*	VIF	*β*	*SE*	VIF	*β*	*SE*	VIF
Strategic inertia	−0.252^***^	0.055	1.036				−0.250^***^	0.058	1.264			
Common humanity				0.335^***^	0.077	1.837	0.396^*^	0.077	1.902	0.247^**^	0.073	1.837
Self-kindness				0.281^***^	0.070	1.485	0.160^*^	0.074	1.732	−0.485^***^	0.066	1.485
Mindfulness				−0.217^**^	0.065	1.650	−0.145^*^	0.065	1.762	0.286^***^	0.061	1.650
Firm size	−0.079^**^	0.026	1.219	−0.075^**^	0.025	1.240	−0.056^*^	0.025	1.279	0.076^**^	0.024	1.240
Firm age	0.125^*^	0.060	1.204	−0.02	0.065	1.481	−0.035	0.063	1.485	−0.059	0.061	1.481
Executive age	−0.070	0.056	1.067	−0.112^*^	0.056	1.170	−0.107	0.055	1.171	0.022	0.053	1.170
*R*^2^	0.099			0.159			0.205			0.209		
*F*	8.961			10.205			11.933			14.312		

^*^p < 0.05, ^**^p < 0.01, ^***^p < 0.001.

The mediating effects were tested using Model 4 of the PROCESS macro (version 3.5) in SPSS 26.0 with the bootstrap method (Hayes, 2013). As shown in Table 5, strategic inertia significantly mediates the relationship between common humanity and firm performance, with an indirect effect of −0.065 (95% CI = [−0.136, −0.003]). It also mediates the relationship between mindfulness and firm performance, with an indirect effect of −0.023 (95% CI = [−0.102, −0.011]). Furthermore, strategic inertia mediates the relationship between self-kindness and firm performance, with an indirect effect of 0.049 (95% CI = [0.009, 0.108]). These results support Hypothesis H5.

**Table 5:** PROCESS mediation test results.

Path	Total effect			Direct effect			Indirect effect		
	Effect	SE	95% IC	Effect	SE	95% IC	Effect	SE	95% IC
Common humanity → Strategic inertia → Firm performance	0.324	0.098	[0.013, 0.395]	0.388	0.075	[0.253, 0.551]	−0.065	0.033	[−0.136, −0.003]
Self-kindness → Strategic inertia → Firm performance	0.291	0.086	[−0.043, 0.067]	0.242	0.073	[0.102, 0.394]	0.049	0.026	[0.009, 0.108]
Mindfulness → Strategic inertia → Firm performance	0.035	0.074	[0.043, 0.332]	0.085	0.063	[−0.021, 0.224]	−0.050	0.023	[−0.102, −0.011]

To further assess the robustness of the mediation effects, we conducted an additional test using structural equation modeling (SEM) with the bootstrap method. Compared to regression analysis using observed variables, SEM allows for the simultaneous estimation of measurement errors at the latent variable level, thereby providing a more stringent test of mediation. As shown in Table 6, the bootstrap test results under the SEM framework are consistent with those from the PROCESS analysis. Specifically, common humanity exerts a significant indirect effect on firm performance through strategic inertia (indirect effect = −0.054, Bias-corrected 95% CI = [−0.109, −0.019]). Self-kindness exerts a significant positive indirect effect on firm performance through strategic inertia (indirect effect = 0.135, Bias-corrected 95% CI = [0.035, 0.271]). Mindfulness exerts a significant indirect effect on firm performance through strategic inertia (indirect effect = −0.042, Bias-corrected 95% CI = [−0.110, −0.005]). None of the bias-corrected or percentile confidence intervals for these paths includes zero, indicating that all mediation effects are statistically significant. Overall, the results from both the PROCESS regression analysis and the SEM bootstrap test are highly consistent in terms of direction and significance, demonstrating the robustness of the mediating role of strategic inertia between the dimensions of self-compassion and firm performance. Therefore, Hypothesis H5 is further supported.

**Table 6:** SEM bootstrap mediation test results.

Path	Indirect effect	SE	Bias-corrected 95%CI			Percentile 95%CI		
			Lower	Upper	*P*	Lower	Upper	*P*
Common humanity → Strategic inertia → Firm performance	−0.054	0.023	−0.109	−0.019	0.005	−0.099	−0.011	0.013
Self-kindness → Strategic inertia → Firm Performance	0.135	0.059	0.035	0.271	0.004	0.031	0.262	0.006
Mindfulness → Strategic inertia → Firm performance	−0.042	0.027	−0.110	−0.005	0.017	−0.106	−0.003	0.024

## 5 Conclusion and implications

Based on the upper echelons theory, this study empirically investigates the relationships among executives’ self-compassion, strategic inertia, and firm performance in the architectural design industry in China. The data analysis indicated that different dimensions of self-compassion exert different effects on strategic inertia: Higher levels of common humanity and mindfulness are significantly positively correlated with strategic inertia, whereas a higher level of self-kindness is significantly negatively correlated with inertia. Strategic inertia partially mediated the relationship between self-compassion and firm performance. These findings provide new evidence for understanding how deep-seated psychological traits of executives influence strategic organizational outcomes through cognitive processes.

This study makes three contributions to existing literature. First, it deepens cognitive understanding of the psychological antecedents of strategic inertia. Moving beyond traditional demographic variables, the study reveals the differentiated pathways through which the distinct dimensions of the integrative psychological construct of self-compassion affect strategic inertia, thereby helping to unpack the “black box” of “executive traits → cognitive filtering → strategic choice.” Second, it extends the application boundaries of the self-compassion theory. While prior research on self-compassion has primarily focused on the individual or team level, this study, grounded in the upper echelons theory, is among the first to extend its application to the corporate strategy level, confirming its explanatory power for organization-level strategic phenomena and bridging micro-level psychology with a macro-level strategy. Third, it enriches the research lineage of the upper echelons theory. The results demonstrate that, beyond classic traits such as the Big Five personality factors, core psychological capacities closely tied to cognitive-affective processes such as self-compassion are also significant predictors of strategic decision-making tendencies.

The following managerial implications are proposed based on the empirical findings. First, simplistic judgments of the merits of executives’ psychological traits should be avoided. For instance, while this study finds that high common humanity may be positively associated with strategic inertia in dynamic industries, it does not imply that firms should seek “low-empathy” leaders. A more pragmatic recommendation is that boards or governance bodies should recognize the potential cognitive tendencies associated with such traits in industries characterized by high uncertainty and a frequent need for strategic change. They can mitigate possible cognitive blind spots by building diverse top management teams, establishing challenger roles, or improving strategic debate mechanisms rather than simply excluding leaders with these traits. Second, organizations can systematically foster self-kindness. Through interventions such as mindfulness training and leadership development programs, firms can enhance their executives’ capacity for self-acceptance and emotion regulation when facing setbacks, which helps reduce defensive cognition and increase strategic flexibility. Third, organizations should optimize their strategic information processing procedures. Acknowledging that executive cognition acts as a filter for strategic decisions, firms should establish institutionalized internal and external environmental scanning mechanisms and introduce multi-perspective strategic analysis processes to mitigate the negative impact of individual leaders’ cognitive limitations on organizational adaptability.

This study has several limitations that indicate clear directions for future research. First, two-wave data have inherent limitations in establishing causal inferences among the variables. Future studies could adopt longitudinal panel designs or case study methods to effectively reveal the dynamic evolutionary pathways between the variables. Second, relying solely on subjective reports of firm performance may introduce common method bias. Subsequent research could improve these measurements by incorporating objective financial indicators or multisource evaluation data. Meanwhile, the assessment of psychological constructs, such as self-compassion, could also benefit from multi-method approaches to enhance validity. Third, this study employed snowball sampling, a non-probability sampling method, which may limit the representativeness of the sample and constrain the external validity of the findings. Moreover, the sample was concentrated in China’s architectural design industry, where a highly dynamic context may amplify the effects of certain traits. Therefore, the generalizability of the conclusions needs to be further tested across different cultural backgrounds, industry types (e.g., stable industries), and organizational sizes to clarify the theoretical boundaries. Finally, the theoretical model could be further extended and deepened, and future work could explore the composition and interactive effects of self-compassion at the team level and introduce contextual variables such as environmental dynamism and board monitoring as moderators to build a more contextualized theoretical framework. Future research could employ probability sampling or other systematic sampling methods to verify and extend the conclusions of this study.

## Data Availability

The raw data supporting the conclusions of this article will be made available by the authors, without undue reservation.

## References

[ref1] Ashford S. Lanaj K. Jennings R. Krishnan S. (2022). When leader self-care begets other care: leader role self-compassion and helping at work. J. Appl. Psychol. 107, 1543–1560. doi: 10.1037/apl0000957, 34647780

[ref2] Ashton M. C. Lee K. (2021). On the relations between HEXACO agreeableness (versus anger) and honesty-humility. Scand. J. Psychol. 62, 887–894. doi: 10.1111/sjop.12772, 34562027

[ref3] Auh S. Menguc B. Katsikeas C. S. Jung Y. S. (2019). When does customer participation matter? An empirical investigation of the role of customer empowerment in the customer participation–performance link. J. Mark. Res. 56, 1012–1033. doi: 10.1177/0022243719866408

[ref4] Back P. Rosing K. Kraft P. S. Dickler T. A. Bausch A. (2020). Ceos' temporal focus, firm strategic change, and performance: insights from a paradox perspective. Eur. Manag. J. 38, 884–899. doi: 10.1016/j.emj.2020.04.009

[ref9001] Bingham C. B. Davis J. P. (2012). Learning sequences: Their existence, effect, and evolution. Acad. Manag. J. 55, 611–641. doi: 10.5465/amj.2009.0331

[ref5] Brislin R. W. (1970). Back-translation for cross-cultural research. J. Cross-Cult. Psychol. 1, 185–216. doi: 10.1177/135910457000100301

[ref6] Bunjak A. Bruch H. Černe M. (2022). Context is key: the joint roles of transformational and shared leadership and management innovation in predicting employee IT innovation adoption. Int. J. Inf. Manag. 66:102516. doi: 10.1016/j.ijinfomgt.2022.102516

[ref7] Carpenter M. A. (2000). The price of change: the role of CEO compensation in strategic variation and deviation from industry strategy norms. J. Manage. 26, 1179–1198. doi: 10.1016/S0149-2063

[ref8] Chiu S. S. Pathak S. Hoskisson R. E. Johnson R. A. (2020). Managerial commitment to the status quo and corporate divestiture: can power motivate openness to change? Leadersh. Q. 33:101459. doi: 10.1016/j.leaqua.2020.101459

[ref10] Dodson S. J. Heng Y. T. (2022). Self-compassion in organizations: a review and future research agenda. J. Organ. Behav. 43, 168–196. doi: 10.1002/job.2556

[ref11] Ferradás Canedo M. M. Freire Rodríguez C. Prada Palmeiro L. Núñez J. C. Rodríguez Martínez S. (2022). Coping profiles and their relationship with self-compassion in childhood. Psicothema 1, 41–48. doi: 10.7334/psicothema2021.269, 35048894

[ref12] Finkelstein S. Hambrick D. C. (1990). Top-management-team tenure and organizational outcomes: the moderating role of managerial discretion. Admin. Sci. Q. 35, 484–503. doi: 10.2307/2393314

[ref13] Giancola M. Palmiero M. Piccardi L. D'Amico S. (2021). The contribution of planning to real-world creativity: the moderating role of agreeableness. Think. Skills Creat. 41:100890. doi: 10.1016/J.TSC.2021.100890

[ref14] Guo X. Fan C. Chen Y. (2025). Executive cognitive styles and enterprise digital strategic change under environmental dynamism: the mediating role of absorptive capacity in a complex adaptive system. Systems 13:775. doi: 10.3390/systems13090775

[ref15] Hambrick D. C. (2007). Upper echelons theory: an update. Acad. Manag. Rev. 32, 334–343. doi: 10.5465/amr.2007.24345254

[ref16] Hambrick D. C. Mason P. A. (1984). Upper echelons: the organization as a reflection of its top managers. Acad. Manag. Rev. 9, 193–206. doi: 10.5465/AMBPP.1982.4976402

[ref17] Hayes A. F. (2013). Introduction to Mediation, Moderation, and Conditional process Analysis: A Regression-based Approach. ed. Todd D. L. (New York: Guilford Press).

[ref18] Hitt M. Keats B. W. Demarie S. (1998). Navigating in the new competitive landscape: building strategic flexibility and competitive advantage in the 21st century. Acad. Manage. Perspect. 12, 22–42. doi: 10.5465/AME.1998.1333922

[ref19] Hortovanyi L. Szabo R. Z. Fuzes P. (2021). Extension of the strategic renewal journey framework: the changing role of middle management. Technol. Soc. 65:101540. doi: 10.1016/j.techsoc.2021.101540

[ref20] Judge T. A. Bono J. E. Ilies R. Gerhardt M. W. (2002). Personality and leadership: a qualitative and quantitative review. J. Appl. Psychol. 87, 765–780. doi: 10.1037/0021-9010.87.4.765, 12184579

[ref21] Kwee M. (1995). Wherever you go, there you are: mindfulness meditation in everyday life. Behav. Res. Ther. 33, 996–996. doi: 10.1016/0005-7967(95)90133-7

[ref22] Leicht-Deobald U. Huettermann H. Bruch H. Lawrence B. S. (2021). Organizational demographic faultlines: their impact on collective organizational identification, firm performance, and firm innovation. J. Manage. Stud. 58, 2240–2274. doi: 10.1111/joms.12747

[ref23] LePine J. A. Van Dyne L. (2001). Voice and cooperative behavior as contrasting forms of contextual performance: evidence of differential relationships with big five personality characteristics and cognitive ability. J. Appl. Psychol. 86, 326–336. doi: 10.1037/0021-9010.86.2.326, 11393444

[ref24] Levinthal D. A. March J. G. (1993). The myopia of learning. Strateg. Manage. J. 14, 95–112. doi: 10.1002/smj.4250141009

[ref25] Lian Y. He X. (2015). CEO openness, strategic inertia, and organizational performance: an empirical analysis based on Chinese listed companies. J. Manage. Sci. China 18, 1–19.

[ref26] Liao Y. H. Stead G. B. Liao C. Y. (2021). A meta-analysis of the relation between self-compassion and self-efficacy. Mindfulness 12, 1878–1891. doi: 10.1007/s12671-021-01626-4

[ref27] Matthew S. K. Edward J. Z. (2001). How organizational resources affect strategic change and performance in turbulent environments: theory and evidence. Organ. Sci. 12, 632–657. doi: 10.1287/orsc.12.5.632.10088

[ref28] Maurer T. J. Allen D. G. Weiss E. M. (2018). “The role of managerial derailment in adaptive performance failure,” in Adaptation in the Workplace, (Cham: Springer), 145–168.

[ref29] Neff K. D. (2003). The development and validation of a scale to measure self-compassion. Self Identity 2, 223–250. doi: 10.1080/15298860309027

[ref30] Nwafor C. N. Nwafor O. Z. Omenihu C. M. Abdrakhmanova M. (2025). Do CEO traits matter? A machine learning analysis across emerging and developed markets. Adm. Sci. 15:268. doi: 10.3390/admsci15070268

[ref31] Ocasio W. (1997). Towards an attention-based view of the firm. Strateg. Manag. J. 18, 187–206. doi: 10.1002/(SICI)1097-0266(199707)18:1+<187::AID-SMJ936>3.0.CO;2-

[ref32] Richard P. J. Devinney T. M. Yip G. S. Johnson G. (2009). Measuring organizational performance: towards methodological best practice. J. Manag. 35, 718–804. doi: 10.2139/ssrn.814285

[ref33] Rosenthal S. A. Pittinsky T. L. (2006). Narcissistic leadership. Leadersh. Q. 17, 617–633. doi: 10.1016/j.leaqua.2006.10.005

[ref34] Salancik G. R. Pfeffer J. (1978). A social information processing approach to job attitudes and task design. Adm. Sci. Q. 23, 224–253. doi: 10.2307/2392563, 10307892

[ref9002] Stryker S. Burke P. J. (2000). The past, present, and future of an identity theory. Soc. Psychol. Q. 63, 284–297. doi: 10.2307/2695840

[ref37] Tian H. Liu H. Wang X. (2025). More open, more innovative? CEOs’ openness in promoting digital transformation. Curr. Psychol. 44, 5638–5653. doi: 10.1007/s12144-025-07555-y

[ref38] Tripsas M. Gavetti G. (2000). Capabilities, cognition, and inertia: evidence from digital imaging. Strateg. Manag. J. 21, 1147–1161. doi: 10.1002/1097-0266(200010/11)21:10/11<1147::AID-SMJ128>3.0

[ref39] Tuggle C. S. Borgholthaus C. J. Harms P. D. O'Brien J. P. (2024). Setting the tone to get their way: an attention-based approach to how narcissistic ceos influence the board of directors to take more risk. Strateg. Manag. J. 45, 2095–2121. doi: 10.1002/smj.3610

[ref40] Wiersema M. F. Bantel K. A. (1992). Top management team demography and corporate strategic change. Acad. Manag. J. 35, 91–121. doi: 10.5465/256474

[ref41] Williamson O. E. (1985). The economic Institutions of Capitalism: Firms, Markets, Relational Contracting. New York: Free Press.

[ref42] Xue K. Wu Y. Wang Z. (2022). Female CEOs, risk-taking, and corporate strategic change. Soft Sci. 36, 1–11. doi: 10.13956/j.ss.1001-8409.2022.11.17

[ref43] Yu D. K. Zhu K. Xiao H. (2025). The relationship between CEO intellectual traits and enterprise digital transformation. Science Technol Progress Policy 11, 127–137. doi: 10.6049/kjjbydc.2024010122

[ref44] Zhang Y. Rajagopalan N. (2010). Once an outsider, always an outsider? CEO origin, strategic change, and firm performance. Strateg. Manag. J. 31, 334–346. doi: 10.1002/smj.812

